# Influence of the implant-abutment connection on the ratio between height and thickness of tissues at the buccal zenith: a randomized controlled trial on 188 implants placed in 104 patients

**DOI:** 10.1186/s12903-020-1037-5

**Published:** 2020-02-17

**Authors:** Davide Farronato, Pietro Mario Pasini, Mattia Manfredini, Cristian Scognamiglio, Andrea Alain Orsina, Marco Farronato

**Affiliations:** 1 Department of Medicine and Surgery, School of Dentistry, University of Insubria, Varese, Italy; 2 Private Practice, corso Europa 10, 20122 Milan, Italy; 3 Private Practice, corso della Vittoria 744, 21042 Caronno Pertusella, Varese, Italy; 4 Fondazione IRCCS Cà Granda Ospedale Maggiore Policlinico, Department of Orthodontics, School of Dentistry, Milan, Italy

**Keywords:** Dental implants, Aesthetics, Tissue height, Tissue thickness, Conical implant–abutment connection, Platform switching

## Abstract

**Background:**

To compare tissue response to two implant systems, featuring internal hexed connections with different designs.

**Methods:**

Patients enrolled in this randomized controlled trial were assigned to two groups. In Group 1, patients were treated with implants with a 5° conical internal hexed connection (Anyridge®, MegaGen, South Korea). In Group 2, patients were treated with implants with an internal hexed connection (Core®, Kristal, Italy). After implant placement and a provisionalisation period of 12 months, impressions were taken, stone casts were poured and digitised with a desktop scanner (D700®, 3Shape, Copenhagen, Denmark). In a digital environment, for each fixture, two values were collected at the buccal zenith: the height of the peri-implant mucosa (mucosal height; MH), calculated from the vestibular shoulder of the implant analogue to the upper gingival margin of the supra-implant tissue; and the width of the peri-implant mucosa (mucosal thickness; MT), calculated from the vestibular shoulder of the analogue to the external mucosa point perpendicular to the implant major axis. The mean and standard deviation for MH and MT, as well as their ratios, were calculated for each group; the sectors in which the implants were placed were also considered. Finally, correlation between MH, MT, connection type and sector was assessed by Pearson’s correlation coefficient, with significance level set at 0.05, and a confidence interval (CI) set at 95%.

**Results:**

Data deriving from 188 implants placed in 104 patients were evaluated. The mean MH values were 3.32 (± 0.12) and 2.70 (± 0.16) mm for Groups 1 and 2, respectively. The mean MT values were 4.37 (± 0.16) and 3.93 (± 0.18) mm for Groups 1 and 2, respectively. Group 1 showed higher MH and MT values and a better ratio (1.50 ± 0.88) than Group 2 (1.81 ± 1.20). The MH, MT and MH/MT ratio were significantly influenced both by sector (*p* = 0.015) and group (*p* = 0.047).

**Conclusions:**

Within the limits of this study, the 5° connection implants supported a more extended tissue height and thickness at the buccal zenith, and a better ratio between them.

**Trial registration:**

This study was retrospectively registered in Clinicaltrials.gov, with number NCT04160689, dated 13/11/2019.

## Background

Implant rehabilitation is a daily practice in dentistry, and patients often have high expectations of this treatment from a functional and aesthetic point of view [1, 2]. In the past, the main parameter for the success of implant therapy was osseointegration and its maintenance in the long term [3]. Today, the stability of osseointegration over time remains crucial, but attention has shifted also to the aesthetic outcome of implant therapy. This is particularly important for implant rehabilitation in the anterior areas, where the final aesthetic result is key; consequently, many authors have investigated this matter, to establish guidelines that could help to achieve predictable and repeatable aesthetic outcomes [4, 5].

Unfortunately, when substituting a natural tooth with an implant-supported crown, the surrounding tissues are subject to modifications [6] that might deteriorate the smile harmony.

In fact, as unequivocally demonstrated by the literature, after tooth extraction, a physiological mechanism of bone resorption is triggered [7, 8]; this resorption, concentrated in the first 4–6 months after extraction, is followed by soft tissues recession, and can therefore compromise the aesthetic result of the implant therapy [9]. Such tissue contraction can be particularly marked in the anterior aesthetic area of the maxilla, where the thin bundle bone is mainly vascularized by the periodontal ligament, and more prone to resorption [9, 10]; this can represent a challenge for the clinician, exspecially in the case of immediate implant placement [11, 12].

Implant manufacturers are therefore trying to increase the biological compatibility of their systems, to compensate for the changes occurring to the hard and soft tissues around the implant. They invest substantial resources to reduce the bone remodelling that occurs after implant placement and functionalization [13]; at the same time, a significant new chapter in implantology is represented by the interaction of soft tissues with the fixture. As a consequence, new parameters might be kept in consideration, such as the height and width of the peri-implant soft tissues. This would allow estimating the grade of acceptance of the biological system and, consequently, the ability not to impact the original shape and appearance of the gum.

Nozawa published one of the first articles investigating these parameters [14]. This 2006 study investigated the volume of soft tissue around internal hexagon implants with a flat-to-flat connection [14]. Fourteen patients installed with single implants were evaluated. After an average time of 3 months, the height/ thickness ratio of the peri-implant tissues amounted to 1/1.5. The authors speculated that tissue width could regulate changes in tissue height [14]. This ratio may represent the volumetric tendency to maturation of the peri-implant soft tissues. In particular, the horizontal thickness at the implant–abutment connection may be a strong influencing parameter, able to prevent recession during tissue maturation and remodelling [14]. In other words, following this concept, the authors emphasized that it is essential to have a sufficient tissue thickness, in order to get adequate tissue height and therefore an excellent aesthetic integration [14].

However, implant companies produce different designs at the abutment connection, with different degrees and angulations, which may cause different tissue adaptations.

One of the most discussed differences between two-piece implants is the connection [15]. Wang’s studies on the microgap [15] between two-piece implants added important knowledge to the literature on the implant–abutment connection. Different researches have investigated bone response to various implant–abutment connections [16, 17] and abutment designs. Among these studies, platform switching, i.e. the mistmatch between the implant platform and the abutment diameter, has emerged as an interesting concept [18], proving a certain efficacy in preserving bone [19] and soft tissue levels [20]; however, still the role played by the connection on soft tissue maturation remains unclear. Nevertheless, many studies are still investigating the topic and suggesting different approaches, such as the use of one-piece implants [21] or the employment of different abutment configurations [22]. Unfortunately, Nozawa’s study [14] used only flat-to-flat connection implants, so no comparison has yet been produced. It might therefore be extremely interesting to study the Nozawa parameters on a wider sample, comparing different connections.

The present article follows a pilot study [23], in which the soft tissue response to 32 single implants was evaluated 1 year after the delivery of provisional restorations, on stone cast models, collecting two values at the buccal site: the mucosal height (MH), calculated from the vestibular shoulder of the implant analogue to the upper gingival margin of the supra-implant tissue; and the mucosal thickness (MT), calculated from the vestibular shoulder of the implant analogue to the external mucosa point perpendicular to the implant major axis. In that pilot study [23] the Nozawa ratio was confirmed, although slightly different. In fact, a mean MH of 3.44 mm (±1.28) was found, with a mean MT of 3.29 (±1.46); therefore, the average of the ratio between MH and MT of the supra-implant mucosa was 1:1.19 (±0.55), with a statistically significant correlation between MH and MT (*p* ≤ 0.01).

However, a wider number of implants was needed for further considerations, and the influence of different connection types had to be considered. Hence, the present study aimed to investigate the influence of the implant–abutment connection on the ratio between height and thickness of tissues at the buccal zenith. The area under analysis should be the most critical for the recession risk. To investigate it, the soft tissue response to two implant systems, featuring internal hexed connections with different degrees (5° versus 45°) was evaluated, in accordance with Nozawa’s parameters. The hypothesis under examination is that different connections may result in different tissue responses or adaptations.

## Methods

### Study design

The present study was designed as a randomized controlled trial. During the period between November 2011 and March 2013, all partially edentulous patients referred to a single dental centre for treatment with dental implants were considered for inclusion.

Inclusion criteria were:

1. age between 18 and 90 years
2. good systemic health
3. good oral hygiene (achieved through professional oral hygiene sessions twice per year, and daily domestic care)
4. fully healed ridges (minimum of 6 months after extraction)
5. full witten and informed consent to participate in this data collection study, attending all periodic follow-up recall.

Exclusion criteria were:

1. severe medical conditions that could affect periodontal health and peri-implant tissue response
2. lactation
3. pregnancy
4. heavy smoking (more than 20 cigarettes/day)
5. use of drugs correlated to periodontal hypertrophy (anticonvulsants such as phenytoin, phenobarbital, vigabatrin, ethosuximide, topiramate and primidone; calcium channel blockers such as nifedipine, amlodipine, and verapamil; and immunosuppressants such as cyclosporine)
6. bone volume that required augmentation procedures before implant placement, as well as soft tissue graft or any kind of peri-implant tissue engineering.

After the application of inclusion and exclusion criteria, the patients selected for enrolment in the study signed a written informed consent form and were randomly assigned to two different groups. In Group 1, patients were treated with an implant with a 5° conical internal hexed connection (Anyridge®, MegaGen, Gyeongbuk, South Korea). In Group 2, patients were treated with a 45° internal hexed connection (Core®, Bioimplant, Kristal Srl, Trezzano sul Naviglio, MI, Italy). Both implants presented a switching platform design. The randomization was performed using a coin, after the application of the inclusion/ exclusion criteria and before the surgical session. The randomization procedure was applied at each implant even if more implants were planned in the same surgical session.

Implants were positioned in all mouth sectors. The mouth was divided into the upper aesthetic sector (maxillary central and lateral incisors, cuspids, first premolars: sector 1; S1), lower aesthetic sector (mandibular central and lateral incisors, cuspids, first premolars: sector 2; S2), upper posterior sector (maxillary second premolars and molars: sector 3; S3) and lower posterior sector (mandibular second premolars and molars: sector 4; S4). All implants were placed in a staged protocol, in fully healed ridges, for supporting single crowns and/or fixed partial prostheses (maximum four elements).

Full written and informed consent to participate in this study was obtained from each patient, as enlisted in the inclusion criteria. The study was conducted in accordance with the principles stated in the Declaration of Helsinki on clinical research involving human subjects, 1975 (revised in 2008), and was approved by the Ethics Committee of the University of Insubria with number #826–0034086: “Studies on the survival and the surgical-prosthetic success of dental implants: Influence of the implant–abutment connection”. Our present manuscript adheres to CONSORT guidelines. In addition, the study has been registered in a publicly available trial register (Trial registration: Clinicaltrials.gov with number NCT04160689, Registered 13 November 2019 - Retrospectively registered, https://clinicaltrials.gov/ct2/show/NCT04160689).

### Surgical and prosthetic procedures

All implants were placed clinically at the bone level [24]. For each implant, conventional and digital endoral periapical radiographs were performed to determine the marginal bone level position [25]. Radiographs were taken according to the long-cone paralleling technique, using a positioner (KerrHawe x-ray holders) parallel to the implant axis and perpendicular to the cone of rays [26]. Implants that were not placed at the bone level were excluded from the statistical analysis. A provisional screw-retained prosthetic restoration was placed after the osteointegration period and left for 12 months for tissue maturation [27].

**Fig. 1:**
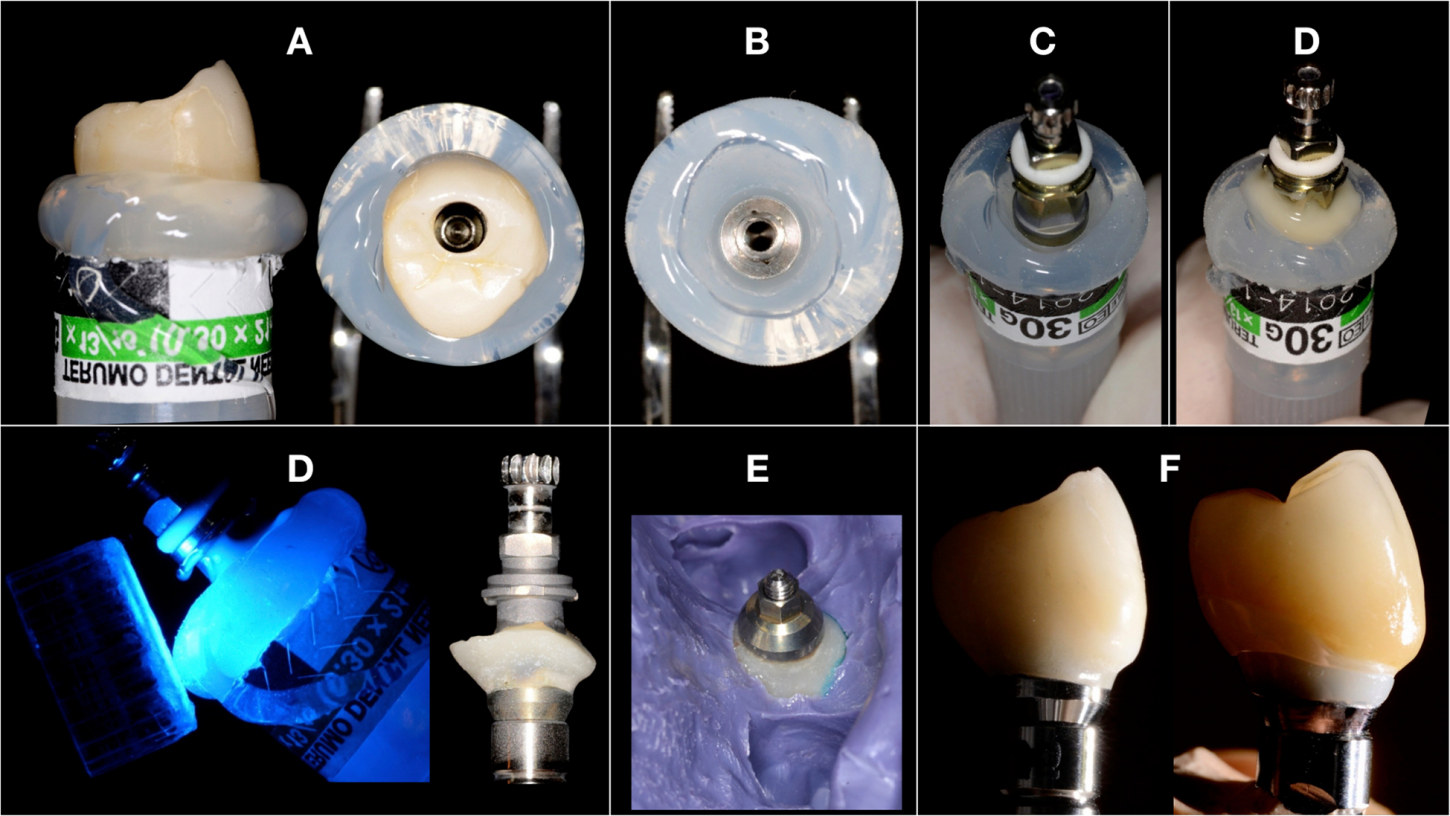
The individual transfer technique was applied in this study. The provisional was connected to an analogue, a silicon impression (Registrado Clear®, Voco, Cuxhaven, Germany) of the assembly was taken (**a**) and, after disconnecting the temporary (**b**), a transfer was set in place, connected to the analogue sitting in the silicone (**c**). Flowable composite was light-cured In the gap between the silicon and the transfer (**d**). The transfers was used for the implant impression (**e**) and reproduced the same emergence as the temporary (**f**)

After 12 months, a silicone impression (Flexitime®, Kulzer GmbH, Hanau, Germany) was taken with an individualised transfer technique. Stone casts were poured. All the dental models included in the study were made of type IV plaster (Fujirock EP®, GC Europe NV, Leuven, Belgium; Modeltypo®, Lascod, Sesto Fiorentino, Italy). The emergence profile of the provisional restoration was reproduced in the final metal-ceramic restoration through the individualised transfer technique (Fig. 1). In case of damaged stone cast, damaged analogue, damaged peri-implant soft tissue or missing stone cast, the patient was excluded from the study. The included models were catalogued according to the implant system used: 5° conical connection (Group 1) and 45° connection (Group 2).

### Measurements and statistical analysis

**Fig. 2:**
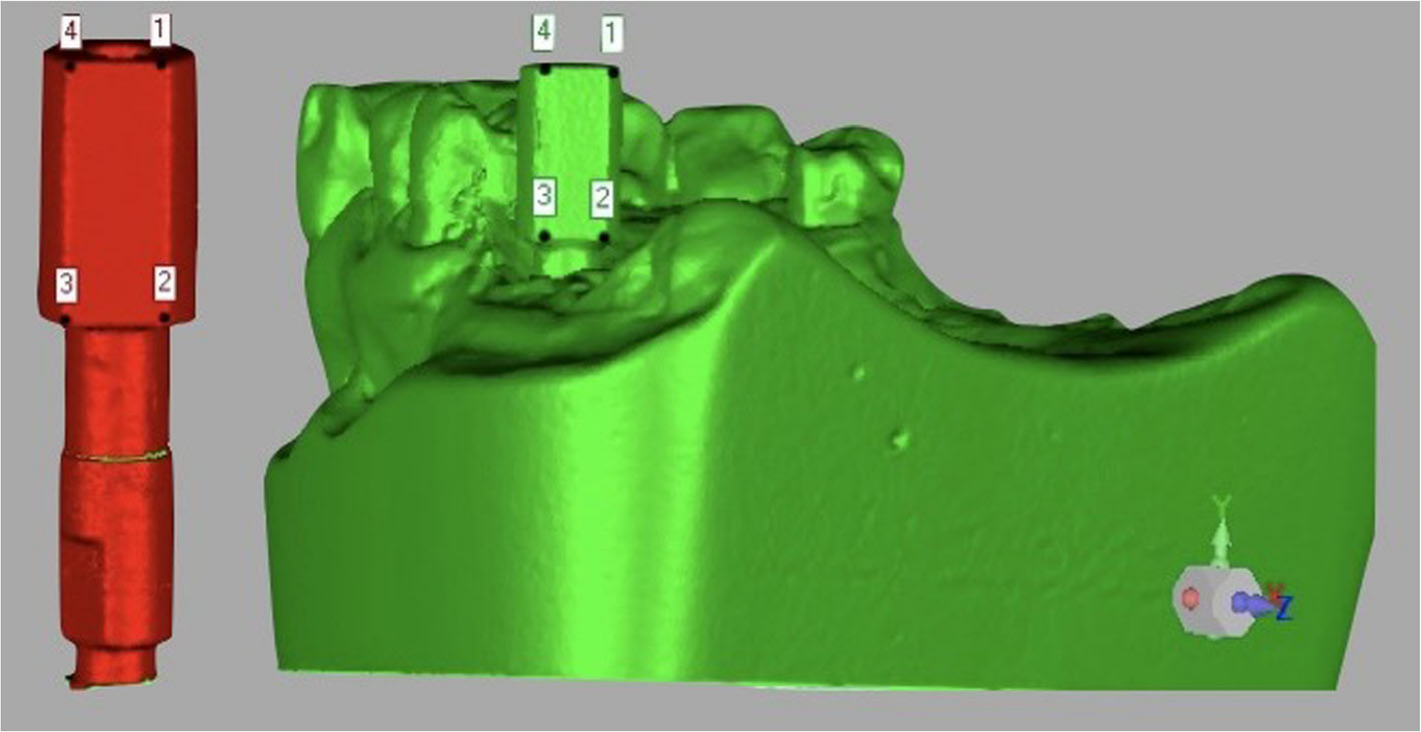
The “three-points registration” function

The scanbodies were screwed on the corresponding implant analogues, then each plaster model was scanned using a desktop machine (D700®, 3Shape, Copenhagen, Denmark). To facilitate the scanning process, a matting spray was applied to the scanbodies (Scan Spray®, Renfert GmbH Company, Hilzingen, Germany). Similarly, each type of analogue was individually connected with the corresponding scanbody and re-scanned. All generated scans were imported into reverse-engineering software (Studio 2012®, Geomagic, Morrisville, NC, USA). Using this software, it was possible to perform the overlapping procedure between each stone cast and the specific analogue/scanbody dataset. The overlaps were performed by two consecutive procedures: first, the ‘three-points registration’ function was used, then three or more points were easily identified on the surface of the scanbodies in each dataset (Fig. 2). This function allowed obtainment of a first alignment of the two 3D surface models. After that, the ‘best fit’ algorithm was applied, for the final superimposition and registration. Overlapping processes were verified, calculating with Geomagic the mean of the distances between the two superimposed models. Overlapped scans were then imported into the GOM Inspect analysis software (GOM Inspect®, GOM GmbH, Braunschweig, Germany). Through this software, cutting sections were performed along the implant axes, and measurements were taken.

**Fig. 3:**
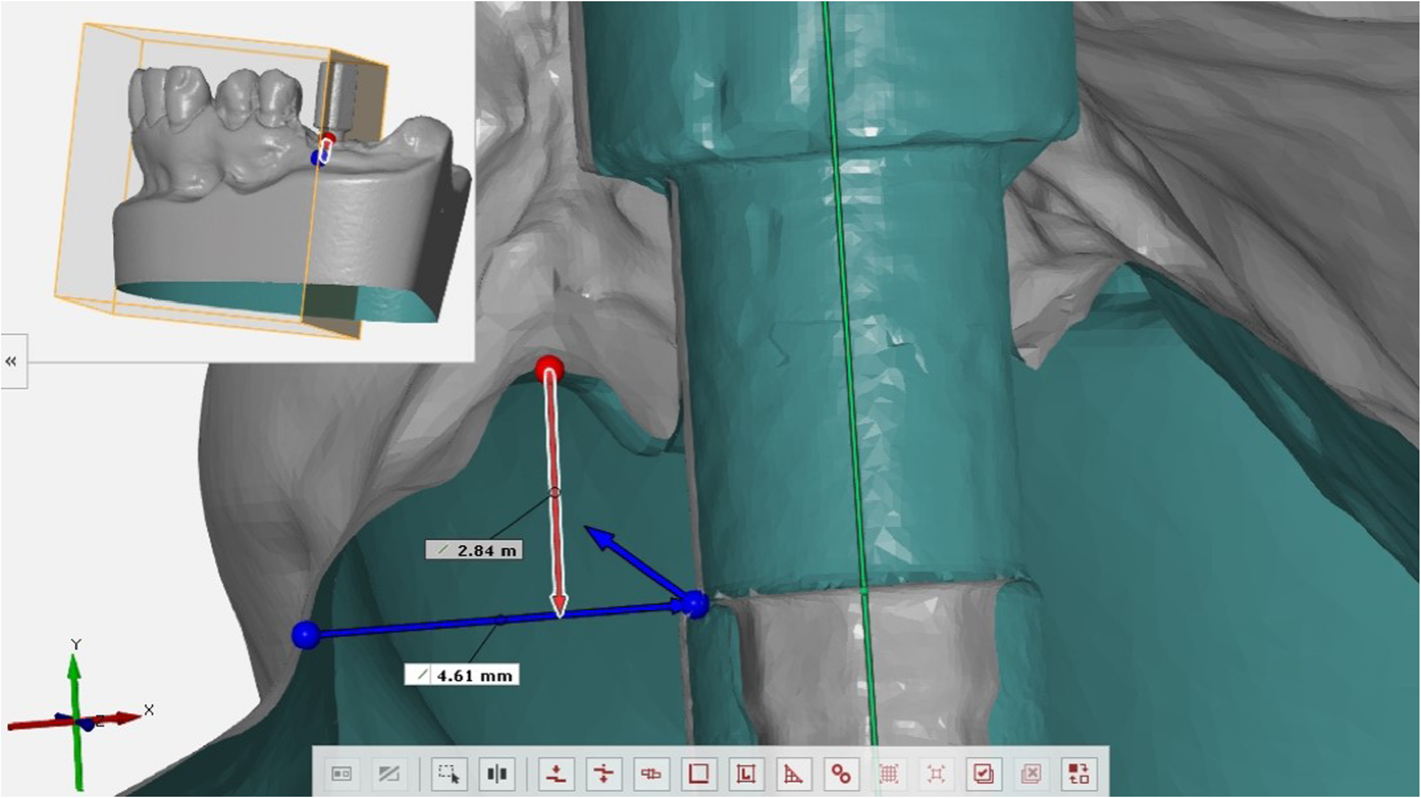
Mucosal height (MH) was calculated from the vestibular shoulder of the analogue to the upper gingival margin of the supra-implant tissue

**Fig. 4:**
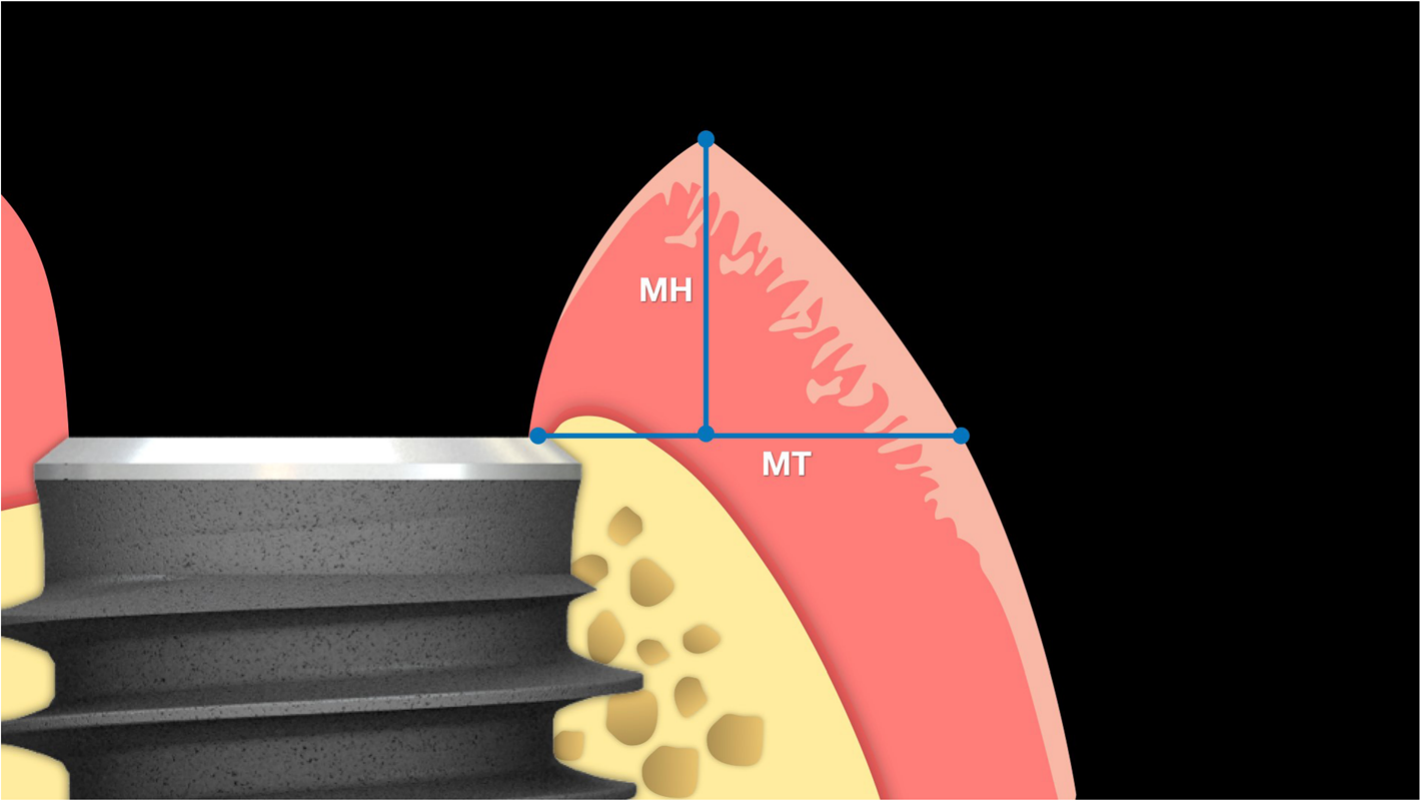
Mucosal thickness (MT) was calculated from the vestibular shoulder of the analogue to the external mucosa point perpendicular to the fixture major axis

For each fixture, two measurements were taken at the buccal zenith: the mucosal height (MH), i.e. height of the peri-implant mucosa and the mucosal thickness (MT) i.e. width of peri-implant mucosa. As reported in a previously published study [23], MH was measured from the vestibular shoulder of the implant analogue to the upper gingival margin of the supra-implant tissue (Figs. 3 and 4). This corresponded to the depth of the implant referred to the most coronal point of the buccal mucosa, measured according to the main implant axis. MT was measured from the vestibular shoulder of the analogue to the external mucosa point, perpendicular to the implant major axis. All measurements were digitally calculated by the 3D software GOM (GOM Italia Srl, Buccinasco, Italy) and registered in an Excel chart. Statistical analysis was performed with SPSS® 17.0 (SPSS Inc., Chicago, IL, USA). The statistical analysis employed a Pearson’s correlation coefficient, with the significance level set at 0.05, to assess the correlation between MH, MT and connection type.

## Results

In total, 104 patients (64 females and 40 males; average age 66, range 43–88 years) were selected for inclusion in this study, and treated with 188 implants. The distribution of the implants was as follows: 29 fixtures (15.4%) were placed in sector S1, 2 (1.1%) in S2, 64 (34.0%) in S3, and 93 (49.5%) in S4 (Table 1). Between the two groups, 125 fixtures (66.5%) with 5° conical connection were placed in Group 1, and 63 fixtures (33.5%) were placed in Group 2.

**Table 1:** Distribution of the implants according to anterior (aesthetic) or posterior areas of upper and lower jaws

Overall				
188 implants	Anterior		Posterior	
Upper	29	15.4%	64	34%
Lower	2	1.1%	93	49.5%
Group 1 (5°)				
125 implants	Anterior		Posterior	
Upper	14	11.2%	47	37.6%
Lower	1	0.8%	63	50.4%
Group 2 (45°)				
63 implants	Anterior		Posterior	
Upper	15	23.8%	17	27%
Lower	1	1.6%	30	47.6%

**Fig. 5:**
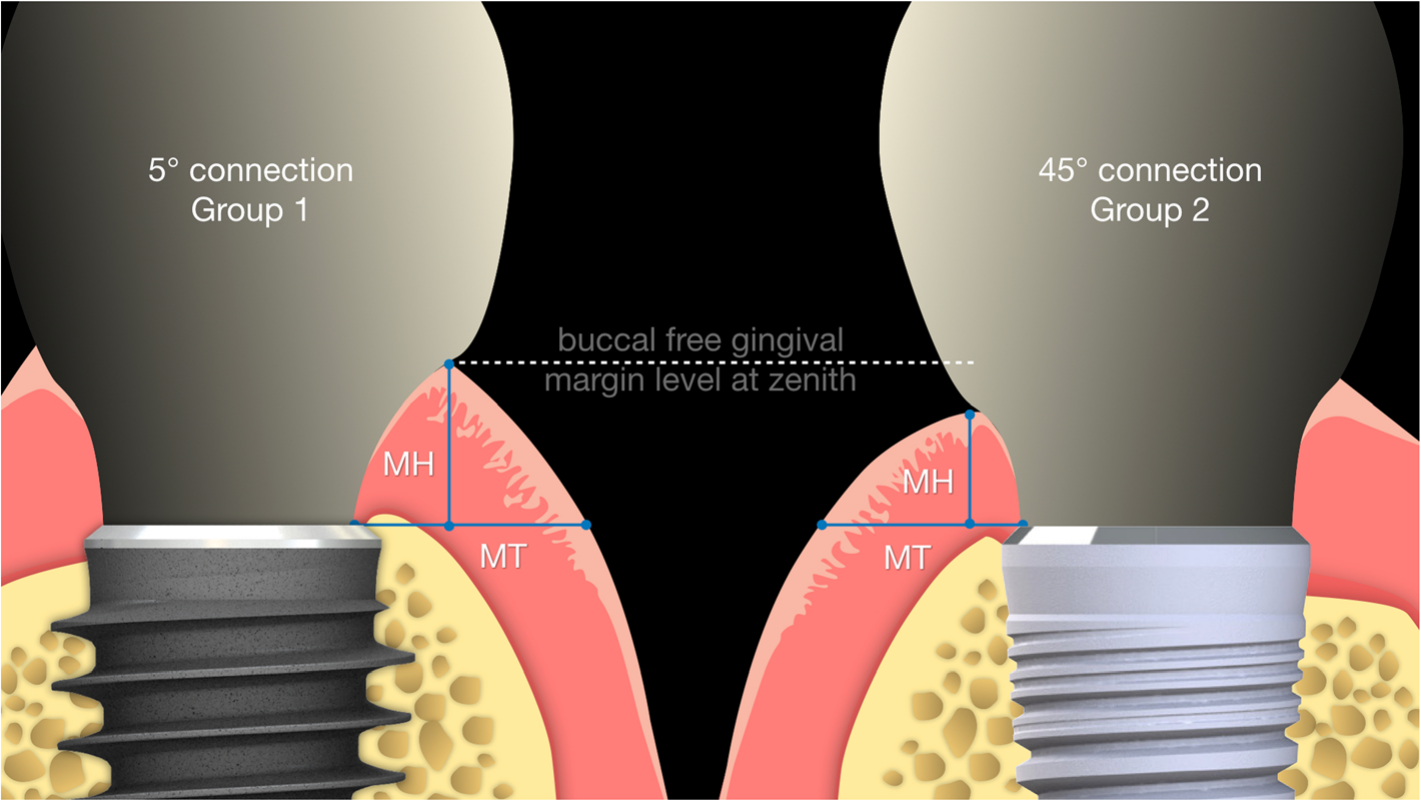
Graphic comparison between Group 1 and Group 2 with average MH and MT proportions

**Fig. 6:**
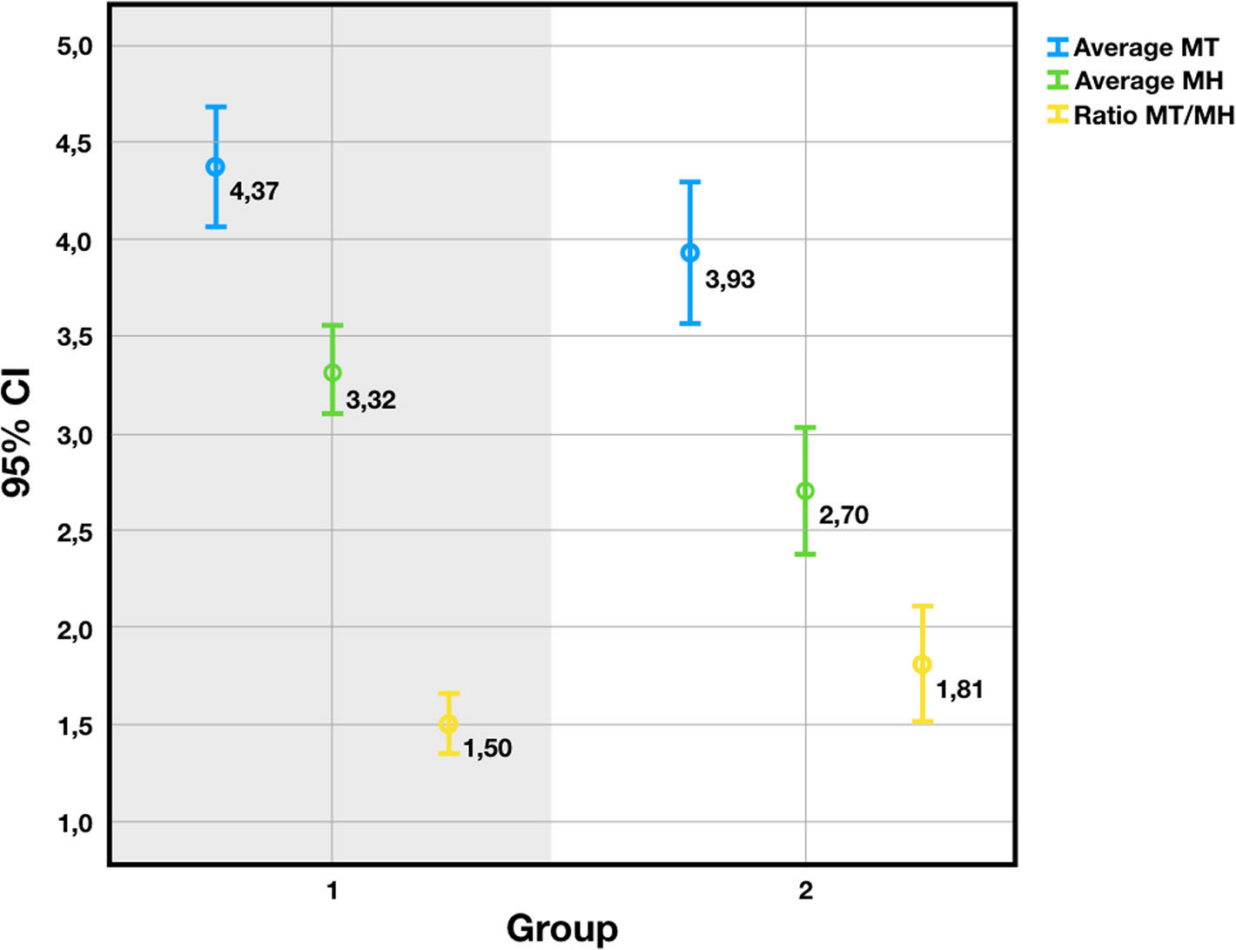
The MT, MH and MT/MH ratio averages depending on the groups

**Fig. 7:**
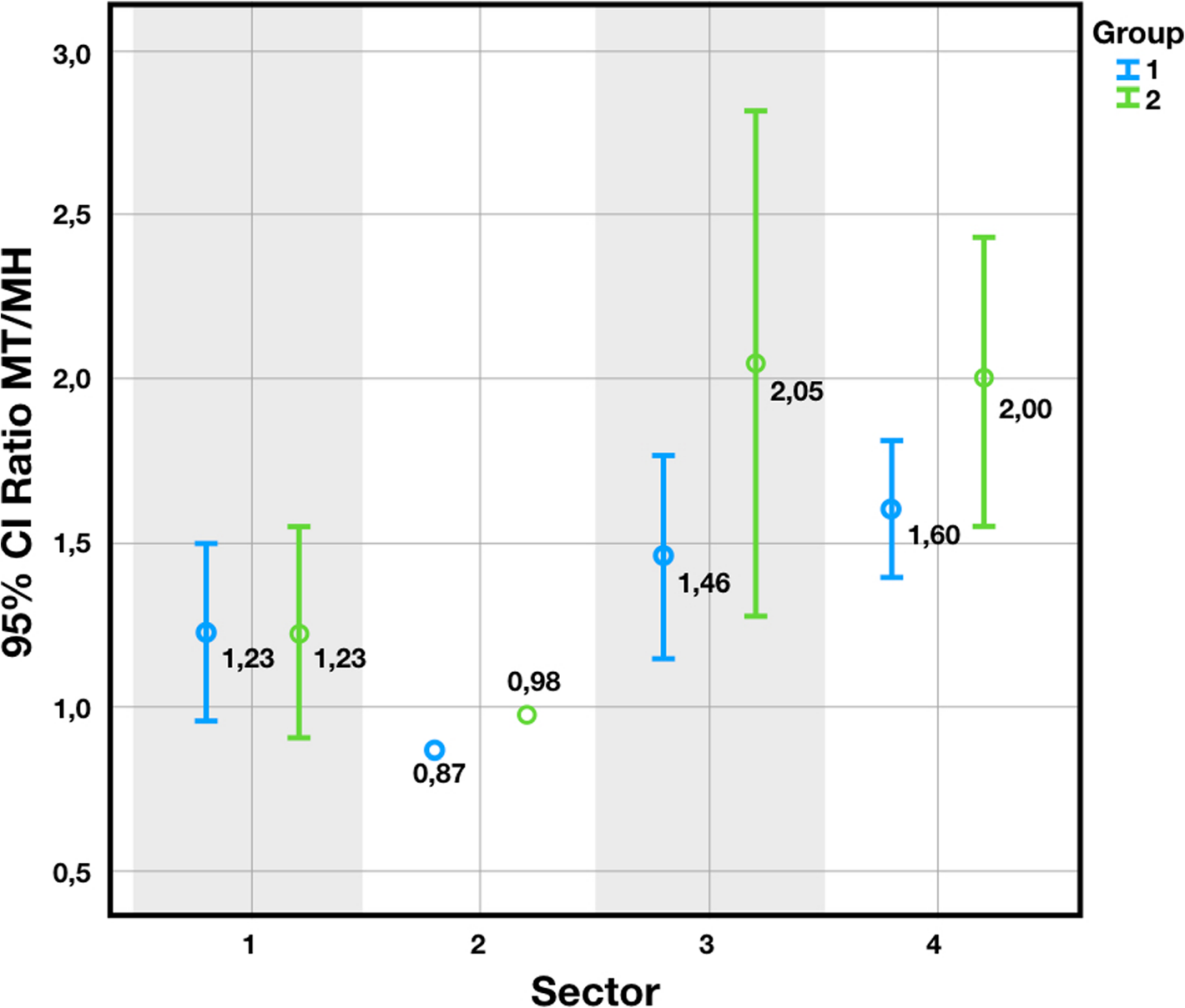
The ratio between MT and MH differs depending on the sector (*p* = 0.015) and the on the groups (*p* = 0.047) (Pearson 2 tailed, 95% conf)

Overall, the average MT was 4.22 mm and the average MH was 3.11 mm. The average MT values were 4.37 (± 0.16) and 3.93 (± 0.18) mm for Groups 1 and 2, respectively. The average MH values were 3.32 (± 0.12) and 2.70 (± 0.16) mm for Groups 1 and 2, respectively (Figs. 5 and 6). According to Nozawa’s criteria [14], the ratio between the facial tissue height and thickness at the connection level (as MT/MH) was calculated. Overall, the obtained average ratio was 1.61 (± 1.0). The ratio was 1.50 (± 0.88) for Group 1, and 1.81 (± 1.20) for Group 2 (Fig. 6). The values were significantly affected by the sector (*p* = 0.015) and group, for which a statistically significant difference emerged (*p* = 0.047) (Pearson two-tailed, 95% CI). The distribution of MT/MH by sector is shown in Fig. 7. The sector, group and gender were not significantly correlated with any other variable (Table 2).

**Table 2:** From the correlation table, it is clear that group and sector significantly influence the MT/MH ratio, whereas no significant correlation is found between sector and group or gender with any other variable

188 Implants	Gender	Group	Sector	MT/MH
Gender	–	0.185	0.614	0.933
Group	0.185	–	0.075	0.047*
Sector	0.614	0.075	–	0.015*
MT/MH	0.933	0.047*	0.015*	–

*Correlation is significant with *p* ≤ 0.05 (Pearson 2 Tailed, 95% Conf)

## Discussion

Achievement of successful implant restoration in the aesthetic area is a focal point in current practice. However, the need still exists to distinguish between implant therapy survival and success criteria. Survival describes just the condition of being in place, but says nothing about the quality of the restoration [28]. On the other hand, success means that the implant results in no pain, mobility, discomfort or infection, is surrounded by stabilised bone and capable of receiving the prosthesis, and has satisfactory aesthetics [29]. To achieve restoration success, many factors are to be taken into account during the surgical stages, as well as in the prosthetic passages [30].

The first stage of successful aesthetic rehabilitation is correct implant placement, starting from the timing [31] of the placement, followed by correct 3D positioning of the fixture [32] even in the case regenerative procedures are necessary [33]. Regarding the correct time for implant placement, a systematic review suggests that, in post-extractive conditions, early placement might be the safest choice to avoid recessions; immediate positioning is subject to a greater variability in outcomes and a higher frequency of recessions > 1 mm of the midfacial mucosa compared to early placement [12]. A more recent study concluded that immediate placement is the best option, but under strict morphological and operator skill–related circumstances; early implant placement with soft tissue healing is otherwise recommended [32]. Regarding the positioning of the fixture, the placement of implants in a correct 3D way is key to an aesthetic treatment outcome regardless of the implant system used. This position is dependent on the planned restoration that the implant will support. The relationship of the position between the implant and the proposed restoration should be based on the position of the implant shoulder, because this will influence the final hard and soft tissue response [34]. The same study stated that an excessively palatal or facial positioning of the fixture might jeopardise the final restorations, due respectively to difficulties of maintenance in the first case or to the risk of soft tissue recession in the second. Moreover, wrong apico-coronal positioning might lead to an undesired bone loss, and incorrect mediodistal placement might lead to a lack of filling of the interdental papilla.

Bone augmentation procedures may be needed if, after the fixture placement, the facial bone thickness is 2 mm or less [35]. As described by Grunder et al., if this amount of bone is not available, part of the buccal bone plate will be lost after remodelling, with the consequence of a high risk of soft tissue recession; such a large amount of bone on the buccal side of the implant head does not exist normally and has to be created with augmentation procedures in almost every aesthetically demanding case [36].

The prosthetic features that should be considered are the type and degree (in terms of angulation) of implant-abutment connection, the shape of the abutment and the shape of the restoration. The presence of platform switching [19] plays a fundamental role in the behaviour of the bony tissues and therefore of the soft tissues. Moreover, Wang [15, 37] reported how significant the effect of the micromotion at the implant–abutment interface might be on the crestal bone.

In 2006, Nozawa investigated the relationship between tissue height and width around internal hexagon implants with a flat-to-flat connection [14]. This study, although based on a limited patient sample and with one only implant system, was the first to investigate this topic.

In a previous pilot work [23], we have demonstrated how Nozawa’s ratios are positively affected by the use of a conical implant-abutment connection. In this work, 32 single Anyridge® implants were placed and after a period of 1 year of provisionalization, precision impressions were taken and stone casts were poured [23]. Then, using an analog method for measurements, two parameters were taken at the buccal site of each fixture, following the Nozawa’s indications: the mucosal height (MH, calculated from the vestibular shoulder of the implant analogue to the upper gingival margin of the supra-implant tissue) and the mucosal thickness (MT, calculated from the vestibular shoulder of the analogue to the external mucosa point perpendicular to the implant major axis). Basically, at the end of this pilot study, a mean MH of 3.44 mm (±1.28) was found, with a mean MT of 3.29 (±1.46) [23]. The Nozawa’s observation were therefore confirmed, with a statistically significant correlation between MH and MT (*p* ≤ 0.01); however, the mean ratio between MH and MT of the supra-implant mucosa amounted to 1:1.19 (±0.55) [23], and was slightly different from that found by Nozawa [14]. The evidence emerging from this pilot study seems to suggest a role of the connection between abutment and implant, in determining the relationship between height and thickness of peri-implant tissues [23].

Following these findings, our present study was designed to assess whether the sector and the implant design, with particular attention for the implant-abutment connection, can affect the tissue trophism and the MT/MH ratio. Based on the type of implants used, patients were assigned to two groups: group 1, patients treated with implants with a 5° conical internal hexed connection (Anyridge®), and group 2, patients treated with implants with a 45° internal hexed connection (Core®). After implant placement and a provisionalisation period of 12 months, impressions were taken, stone casts were poured and digitised with a desktop scanner; then, using digital technologies, the mucosal height (MH) and thickness (MT) were calculated at the buccal side of each fixture. At the end of the study, data deriving from 188 implants placed in 104 patients were evaluated. The mean MH values were 3.32 (± 0.12) and 2.70 (± 0.16) mm for group 1 (implants with a 5° conical internal hexed connection) and 2 (implants with a 45° internal hexed connection) respectively. The mean MT values were 4.37 (± 0.16) and 3.93 (± 0.18) mm for group 1 and 2, respectively. Group 1 showed higher MH and MT values and better ratio (1.50 ± 0.88) than Group 2 (1.81 ± 1.20). The MH, MT and MH/MT ratio were significantly influenced both by sector (*p* = 0.015) and group (*p* = 0.047). In summary, the 5° connection led to thicker and higher tissues related to the implant platform, compared to a 45° connection design. The data should be considered favourable towards the narrower angle, since higher tissue stability in terms of volume might be supposed. In terms of the Nozawa findings, the MT/MH ratio seems to express a measure of healthiness and acceptance of the implant by the surrounding tissues. The free gingival margin depends on the tissue thickness, in relation to blood supply. This last parameter itself, according to Cortellini and Kassab, is a predictive positive factor for recession incidence on the natural tooth [38, 39]. Whenever this does not happen, a loss of verticality might occur, with a consequently higher risk of recession around the implant. It might therefore be supposed that both increased values of MT and MH, and a ratio that lowers in value, corresponds to a minor tendency for the free gingival margin to recession, as confirmed by Kinaia et al. [40]. Consequently, the 5° connection showed more extended MH and thicker MT compared to the 45° connection, reflecting a minor tendency to the undesirable event of recession.

Due to the influence of the sector, a different distribution of implants may be thought to imply a different result in the tissue values. However, no correlation was found between sector and group, so its influence may not be considered significant.

Our present study has limits, because only two implant systems have been investigated and compared, and certainly it would be recommended to extend the analysis to different types of connections and to fixtures with different designs, in order to draw more solid conclusions. In addition, the method used here is indirect digital, by means of conventional impressions subsequently poured in plaster models, digitized with a desktop scanner: this can lead to errors and therefore measurement defects. Moreover, the randomization method used here (coin toss) was a limit too, since there was the possibility of an unbalanced number of participants in the two groups (125 implants in the group 1, versus only 63 implants in the group 2). Finally, in the present study, the choice of abutment height was made in relation to soft tissue thickness; this can represent a limit of the study, because the use of short abutments (≤2 mm) could negatively influence marginal bone loss, irrespective of soft tissue thickness [41, 42]. Further studies are therefore needed to confirm the outcomes emerging from the present one.

## Conclusions

In the present study, 104 patients were allocated into two groups (group 1, implants with a 5° conical internal hexed connection; group 2, implants with a 45° internal hexed connection) and restored with 188 single implants. After 1 year of provisionalisation, impressions were taken, stone casts were poured and digitised with a desktop scanner; then, the mucosal height (MH) and thickness (MT) were calculated at the buccal side of each fixture, using the digital tools of a computer-assisted-design (CAD) software. The mean MH values were 3.32 (± 0.12) and 2.70 (± 0.16) mm for groups 1 and 2, respectively. The mean MT values were 4.37 (± 0.16) and 3.93 (± 0.18) mm for groups 1 and 2, respectively. Group 1 showed higher MH and MT values and a better ratio (1.50 ± 0.88) than group 2 (1.81 ± 1.20). The MH, MT and MH/MT ratio were significantly influenced both by sector (*p* = 0.015) and group (*p* = 0.047). Within the limits of the present study, tissue trophism seems to be sensitive to the sector and the implant connection. Conical connections and platform switching are well known to significantly help in reducing the microgap at the implant connection and stabilising the peri-implant bone and soft tissues. This study went one step further to assess the different effects on the tissues of two different degrees of conicity. In particular, the 5° implant connection showed a significantly higher tissue thickness and height, which might be preferable to the 45° implant, especially in the aesthetic zone. Therefore, the authors recommend using conical 5° connection implants, particularly for the rehabilitation of the anterior area, in order to achieve the best soft tissue response and aesthetic integration.

## Data Availability

The datasets used and/or analysed during the current study are available from the corresponding author on reasonable request.

## Abbreviations

3D: Three-dimensional
CI: Confidence interval
MH: Mucosal height
MT: Mucosal thickness
S: Sector
SD: Standard deviation
